# Additive Effects of Omega-3 Fatty Acids and Thiazolidinediones in Mice Fed a High-Fat Diet: Triacylglycerol/Fatty Acid Cycling in Adipose Tissue

**DOI:** 10.3390/nu12123737

**Published:** 2020-12-04

**Authors:** Kristina Bardova, Jiri Funda, Radek Pohl, Tomas Cajka, Michal Hensler, Ondrej Kuda, Petra Janovska, Katerina Adamcova, Ilaria Irodenko, Lucie Lenkova, Petr Zouhar, Olga Horakova, Pavel Flachs, Martin Rossmeisl, Jerry Colca, Jan Kopecky

**Affiliations:** 1 Laboratory of Adipose Tissue Biology, Institute of Physiology of the Czech Academy of Sciences, Videnska 1083, 142 20 Prague 4, Czech Republic; kristina.bardova@fgu.cas.cz (K.B.); jiri.funda@fgu.cas.cz (J.F.); hensler.michal@gmail.com (M.H.); petra.janovska@fgu.cas.cz (P.J.); katerina.adamcova@fgu.cas.cz (K.A.); ilaria.irodenko@fgu.cas.cz (I.I.); lucie.lenkova@fgu.cas.cz (L.L.); petr.zouhar@fgu.cas.cz (P.Z.); olga.horakova@fgu.cas.cz (O.H.); flachs@biomed.cas.cz (P.F.); martin.rossmeisl@fgu.cas.cz (M.R.); 2 NMR Spectroscopy, Institute of Organic Chemistry and Biochemistry of the Czech Academy of Sciences, Flemmingovo Namesti 542/2, 160 00 Prague 6, Czech Republic; radek.pohl@uochb.cas.cz; 3 Laboratory of Metabolomics, Institute of Physiology of the Czech Academy of Sciences, Videnska 1083, 142 20 Prague 4, Czech Republic; tomas.cajka@fgu.cas.cz; 4 Laboratory of Translational Metabolism, Institute of Physiology of the Czech Academy of Sciences, Videnska 1083, 142 20 Prague 4, Czech Republic; 5 Laboratory of Metabolism of Bioactive Lipids, Institute of Physiology of the Czech Academy of Sciences, Videnska 1083, 142 20 Prague 4, Czech Republic; ondrej.kuda@fgu.cas.cz; 6 Cirius Therapeutics, Kalamazoo, MI 490 07, USA; jcolca@ciriustx.com

**Keywords:** insulin, lipogenesis, obesity, glucose homeostasis, adipocytes

## Abstract

Long-chain n-3 polyunsaturated fatty acids (Omega-3) and anti-diabetic drugs thiazolidinediones (TZDs) exhibit additive effects in counteraction of dietary obesity and associated metabolic dysfunctions in mice. The underlying mechanisms need to be clarified. Here, we aimed to learn whether the futile cycle based on the hydrolysis of triacylglycerol and re-esterification of fatty acids (TAG/FA cycling) in white adipose tissue (WAT) could be involved. We compared Omega-3 (30 mg/g diet) and two different TZDs—pioglitazone (50 mg/g diet) and a second-generation TZD, MSDC-0602K (330 mg/g diet)—regarding their effects in C57BL/6N mice fed an obesogenic high-fat (HF) diet for 8 weeks. The diet was supplemented or not by the tested compound alone or with the two TZDs combined individually with Omega-3. Activity of TAG/FA cycle in WAT was suppressed by the obesogenic HF diet. Additive effects in partial rescue of TAG/FA cycling in WAT were observed with both combined interventions, with a stronger effect of Omega-3 and MSDC-0602K. Our results (i) supported the role of TAG/FA cycling in WAT in the beneficial additive effects of Omega-3 and TZDs on metabolism of diet-induced obese mice, and (ii) showed differential modulation of WAT gene expression and metabolism by the two TZDs, depending also on Omega-3.

## 1. Introduction

The prevention and treatment of obesity and associated pathologies, namely, dyslipidemia, type 2 diabetes, hepatosteatosis, cardiovascular disease and cancer, represent major challenges for the healthcare system. Reflecting on the multiple pathological mechanisms involved, any treatment must include, besides pharmacological interventions, lifestyle changes, including dietary measures. Long-chain n-3 polyunsaturated fatty acids (Omega-3) in the diet, such as eicosapentaenoic acid (EPA; 20:5n-3) and docosahexaenoic acid (DHA; 22:6n-3), exert broad benefits on health (reviewed in [1,2,3,4,5]). These lipids act as natural hypolipidemics, reduce accumulation of hepatic lipids ([6,7]; reviewed in [8]), increase plasma levels of adiponectin ([9]; reviewed in [10]), ameliorate low-grade inflammation in obesity [11] and enhance intestinal fatty acid (FA) oxidation [12,13]. Moreover, Omega-3s reduce adiposity while limiting proliferation of adipocytes in rodent models of obesity [14,15] and could reduce obesity, even in humans [16,17]. Animal experiments document the beneficial effects of Omega-3 on insulin sensitivity and glucose metabolism even under conditions of established insulin resistance. Although Omega-3s cannot reverse insulin resistance in diabetic patients, some intervention studies document their positive effects on glucose homeostasis in prediabetic subjects (reviewed in [18]).

In diet-induced obese mice, we have observed additive effects of Omega-3 and anti-diabetic drugs thiazolidinediones (TZDs) in counteracting obesity and alleviating dyslipidemia, hepatosteatosis, low-grade inflammation of white adipose tissue (WAT) and whole-body insulin resistance. Induction of adiponectin and improvement of insulin sensitivity in muscle were also observed, in the absence of any effect on food consumption [19,20]. Several TZDs were used to improve insulin sensitivity in patients with type 2 diabetes, resulting from the interaction of TZDs with (i) peroxisome-proliferator activated receptor γ (PPARγ), the transcription factor representing the dominant regulator of adipogenesis and fat cell gene expression [21]; and (ii) mitochondrial pyruvate carrier, which is inhibited by TZD binding [22,23]. However, the “classical” TZDs induce side effects that are mediated by PPARγ. Thus, pioglitazone is the only TZD remaining in clinical use [24]. Second-generation, “PPARγ-sparing” TZDs were developed to reduce the adverse side-effects of TZDs. These novel TZDs minimize direct binding to PPARγ [25] but retain the ability to decrease activity of the mitochondrial pyruvate carrier [25,26,27]. Thus, in s recent phase 2b clinical trial, MSDC-0602K decreased fasting glucose, insulin, glycated hemoglobin and markers of liver injury without dose-limiting side effects [28].

Adverse consequences of obesity are reflected in large ectopic accumulations of lipids in extra-adipose tissues. This “lipotoxic” impact depends in part on the insufficient capacity of WAT to buffer plasma non-esterified fatty acid (NEFA) levels. Thus, intrinsic metabolic properties of WAT play a role, namely, in the activity of the futile cycle based on the hydrolysis of triacylglycerols (TAG) and re-esterification of FAs in adipocytes (TAG/FA cycling). This core biochemical activity of WAT is required for fine and fast tuning of plasma NEFA levels [1,29,30,31,32]. Together with in situ FA synthesis (de novo lipogenesis; DNL), TAG/FA cycling is linked to oxidative phosphorylation (OXPHOS), because ATP is required for both of these processes [1,2,33]. Moreover, TAG synthesis requires constant generation of glycerol 3-phosphate. In WAT, its formation occurs mainly via glycolysis and also from precursors other than glucose, i.e., via glyceroneogenesis, whereas direct phosphorylation of glycerol is of a minor significance [29,34,35].

The activity of TAG/FA cycling in WAT is under complex control, as inferred by its positioning at the intersection of various metabolic fluxes in adipocytes (reviewed in [1,2,33]). Our results indicated that beneficial effects of Omega-3 on plasma lipids, glucose homeostasis and liver fat accumulation in diet-induced obese mice were linked to the stimulation of mitochondrial biogenesis [36] and OXPHOS activity [6] in epididymal WAT (eWAT), and probably also TAG/FA cycling in this tissue [37]. TZDs induce TAG/FA cycling in WAT via stimulation of glyceroneogenesis [29,38]. Whether WAT metabolism can be involved in the beneficial systemic effects of the combined intervention using Omega-3 and TZDs has not yet been studied [19,20,39,40].

Therefore, we have tested the effect of the combined intervention using the model of obesity induced by a high-fat (HF) diet in mice. The experiments were conducted similarly as before [19,20,39], except that in addition to the “classical” TZD pioglitazone, a second-generation TZD, namely, MSDC-0602K, was also used. Moreover, the activity of TAG/FA cycling in eWAT was evaluated in vivo, in parallel with the characterization of eWAT gene expression and other selected whole-body phenotypes. Our results support the role of TAG/FA cycling in WAT for the beneficial additive effects of Omega-3s and TZDs on the metabolism of diet-induced obese mice. We showed differential modulation of eWAT gene expression and metabolism by the two TZDs, depending also on Omega-3.

## 2. Materials and Methods

### 2.1. Animals and Dietary Interventions

Experiments were performed similarly as before [13,19,20,39]. Thus, male C57BL/6N mice (Charles River Laboratories, Sulzfeld, Germany) were maintained at 22 °C in a 12 h light–dark cycle (light from 6.00 a.m.) with free access to water and standard chow diet (STD; 3.4% wt/wt as lipids; rat/mouse—maintenance extrudate; Ssniff Spezialdieten GmbH, Soest, Germany). At 3 months of age, mice were randomly assigned (*n* = 8–10) to the HF diet (lipid content, ≈35% wt/wt, mainly corn oil; [19]) or to the following “interventions,” which were based on feeding (i) HF + F, a HF diet supplemented with Omega-3 concentrate (46% DHA, 14% EPA, wt/wt, as TAG; product EPAX 1050 TG; EPAX a.s., Lysaker, Norway), which replaced 15% wt/wt of dietary lipids to achieve a total EPA and DHA concentration of 30 mg/g diet (tocopherol content 0.02% wt/wt); (ii) HF + PIO, a HF diet supplemented with 50 mg pioglitazone/kg diet (Actos; Takeda, Japan); (iii) HF + PIO + F, a HF diet supplemented with both pioglitazone and Omega-3; (iv) HF + MSDC, a HF diet supplemented with 330 mg MSDC-0602K/kg diet (Cirius Therapeutics, USA); and (v) HF + MSDC + F, a HF diet supplemented with both MSDC-0602K and Omega-3. The dose for supplementation of HF diet by pioglitazone and Omega-3 was as before, i.e., under the conditions when the additive effects of the two interventions could be observed [20]. The dose of MSDC-0602K was the same as in the previous studies using this TZD in mice [25,26,41]. Diets were stored at −20 °C, in sealed plastic bags filled with nitrogen. The animals received fresh aliquots of the diet every other day. For the composition of macronutrients and the FA profiles in HF diets (see ESM Table 2 of ref. [19], where cHF and cHF + F represent HF and HF + F, respectively, of the present study). Some mice were maintained on the STD to serve as lean controls. Mice were fed the respective diets for 8 weeks. Body weights were recorded every other week, while 24 h food consumption was measured every week.

Mice were sacrificed in a non-fasted state by cervical dislocation under diethyl ether anesthesia (between 8.00 and 10.00 a.m.). Liver, eWAT and subcutaneous WAT in the inguinal and gluteal region (scWAT) were dissected and snap frozen in liquid nitrogen; ethylenediaminetetraacetic acid (EDTA) treated plasma was collected; and all the samples were stored at −80 °C. The experiments followed the guidelines for the use and care of laboratory animals of the Institute of Physiology of the Czech Academy of Sciences and were approved under protocol number 81/2016.

### 2.2. Biochemical Analysis of Plasma and Tissue Samples

Plasma levels of (i) TAG and total cholesterol were determined using the colorimetric enzymatic assays from Erba Lachema (Brno, Czech Republic), and (ii) NEFAs were assessed with a NEFA-HR(2) kit from Waco Chemicals GmbH (Neuss, Germany). Blood glucose levels were measured by OneTouch Ultra glucometers (LifeScan, Milpitas, CA, USA), and plasma insulin levels were determined by the Sensitive Rat Insulin RIA Kit (Millipore, Billerica, MA, USA). Liver TAG content was estimated in ethanolic KOH tissue solubilisates as before [13].

### 2.3. Glucose Homeostasis

Intraperitoneal glucose tolerance test (GTT) was performed using 1 mg of glucose/g body weight in overnight fasted mice. The homeostatic model assessment of insulin resistance (HOMA) index was calculated as described [20].

### 2.4. Histological and Immunohistological Analysis of eWAT

As described before [6], formalin-fixed paraffin-embedded tissue sections stained by hematoxylin and eosin were used for adipocyte morphometry (600 objects per section were evaluated). A macrophage marker MAC-2/galectin-3 was detected using specific antibodies to calculate a relative density of crown-like structures (CLS). Digital images were captured using Olympus AX70 light microscope and a DP 70 camera (Olympus, Tokyo, Japan). Images were analyzed using NIS Elements software (Laboratory Imaging, Prague, Czech Republic). All histological analyses were performed by a pathologist blinded to dietary groups.

### 2.5. Real-time Quantitative PCR (RT-qPCR)

Total tissue RNA was isolated and gene expression was evaluated as described [15]. Data were normalized [42] to the geometric mean signal of four reference genes (*Eef2*, *Eef1a1*, *Actb* and *Cyphb/Ppib*). Background gene expression levels were defined by the mean Cp value (the cycle at which the fluorescence of a sample rises above the background fluorescence) lower than 30.

Gene abbreviations and an overview of primer sequences are given in Table 1.

### 2.6. In Vivo Evaluation of TAG synthesis and DNL-derived FA in eWAT

TAG synthesis and DNL were characterized using in vivo ^2^H enrichment of TAG similarly to before [33]. Due to the suppression of TAG/FA cycling activity by HF diet (see Results), the period of in vivo ^2^H-incorporation into TAG was extended to 21 days before dissection, whereas the ^2^H_2_O concentration in drinking water was increased to 10% and the intraperitoneal ^2^H_2_O bolus was omitted. This labeling was performed in 8 out of 10 mice per group; 2 mice served as a negative control for ^2^H_2_O analyses. After dissection, lipids from eWAT were extracted similarly as for FAHFA extraction in [43], except that the samples were homogenized in a mixture of citric acid and ethylacetate. Dried organic phase was resuspended in hexane and applied on Discovery DSC-Si SPE tubes (52 μm, 72 Å; MERCK, Darmstadt, Germany). TAG fraction was eluted from SPE tubes with a mixture of hexane and MTBE. Samples were analyzed using either nuclear magnetic resonance (NMR) spectroscopy or liquid chromatography–mass spectrometry (LC–MS).

^1^H and ^2^H-NMR spectroscopy was performed as before [33] using AVANCE III HD 500 MHz system (Bruker Corporation) equipped with ^19^F lock and a 5-mm CP BBO-^1^H&^19^F-^2^H probe. The spectra were analyzed using MestReNova and spectral deconvolution was used in case of ^2^H for integration of signals. The amounts of ^1^H and ^2^H in both glycerol and fatty-acyl moieties of TAG were calculated from the peak area relative to the peak of the pyrazine ^1^H/^2^H standard. Since (i) ^2^H can be incorporated in a glycerol moiety of TAG only before esterification of FA to glycerol, and (ii) glycerol formed during lipolysis in WAT is assumed to be released into the circulation and not converted to glycerol-3-phosphate in situ (reviewed in [1]; see Discussion), TAG positional ^2^H enrichment of the glycerol moiety reflects the rate of TAG synthesis. Enrichment of newly synthesized glycerol-3-phosphate from ^2^H_2_O was assumed to be stoichiometric for all five positional hydrogens regardless of the relative contributions of glycolysis and glyceroneogenesis [34]; however, relative contributions of the individual carbons to the labeling of the glycerol moiety of TAG might differ. Therefore, the ^2^H enrichment of the glycerol moiety was evaluated separately for (i) sn 1 + 3 and (ii) sn 2 carbon. Similarly, ^2^H enrichment of FA methyls in TAG correlates with de novo FA synthesis (DNL) rate. Measurement of fractional TAG/FA cycling drew on previously validated assumptions of glycerol ^2^H-enrichment from body ^2^H-enriched water [34].

Analysis of both TAG species and their FA after hydrolysis was conducted using LC–MS. Details can be found in Supplemental Materials. In total 135 deuterated TAG species were detected; 62 species above basal were considered, representing 98% of lipids. In total, 44 deuterated FA above basal were detected in TAG hydrolysates; however, only 8 of them, which were detected in all the groups, were considered (Supplementary Table S1). In order to characterize FA desaturation index, the product/substrate ratios (FA 14:1/14:0, FA 16:1/16:0 and FA 18:1/18:0) were calculated from peak heights obtained from LC–MS analysis.

### 2.7. Statistical Analysis

Data are presented as means ± SEM. Statistical analysis was performed using GraphPad Prism (Version 8.3.1, 2019). Dallal and Wilkinson’s approximation to Lilliefors’ method was used to test normality of the data and log_10_ transformed when needed. Data were analyzed in two ways: (i) to compare STD with individual HF-based diets, a two-tailed Student’s t-test or Mann–Whitney’s non-parametric test was used; and (ii) to compare HF-based diets with each other (i.e., to reveal the effects of various interventions), one-way ANOVA followed by the Tukey’s post-hoc test or the Kruskal–Wallis non-parametric test with Dunn’s post-hoc test were done. A *p* ≤ 0.05 was considered to be significant. Partial least squares-discriminant analysis (PLS-DA) was performed using MetaboAnalyst 4.0 [44]. Significant outliers (according to Grubb’s test) were excluded from further analyses.

## 3. Results

### 3.1. Parameters of Energy Balance, Adiposity and Lipid Metabolism Markers

**Figure 1:**
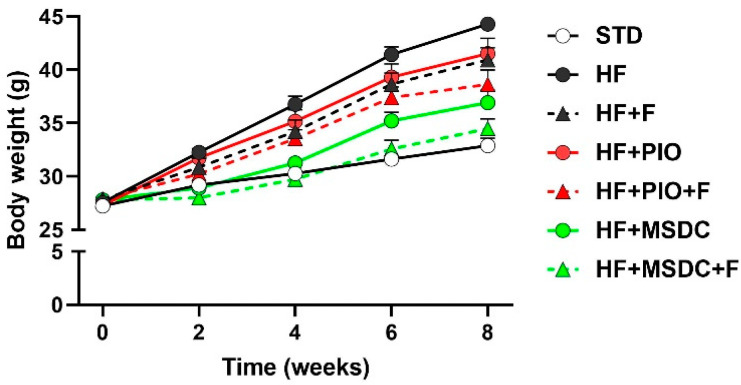
Body weights during dietary interventions. At 3 months of age, subgroups of mice (*n* = 8–10) were fed STD or various high fat (HF) diets for 8 weeks. Data were pooled from two separate experiments (resulting in *n* = 16–19/group). Data are means ± SEMs. For statistical differences at week 8, see Table 2. For designation of dietary groups, see Section 2.1. STD: standard chow diet; HF: high-fat; PIO: pioglitazone.

Body weight was increased by HF diet administration. This obesogenic effect was reduced by all the interventions except HF + PIO and HF + F, which only tended to reduce body weight. The differential effects of diets on body weight became apparent already after 2 weeks of feeding (Figure 1). At the end of the 8-week-study, almost all the interventions significantly prevented body weight gain vs. HF diet (Figure 1 and Table 2): strongest effects for HF + MSDC vs. HF + F and HF + PIO. The combination interventions exerted even stronger effects than individual interventions—the highest (≈2.7-fold) suppression of body weight gain was seen for the HF + MSDC + F mice. None of the HF-based diets affected food consumption (Table 2).

**Figure 2:**
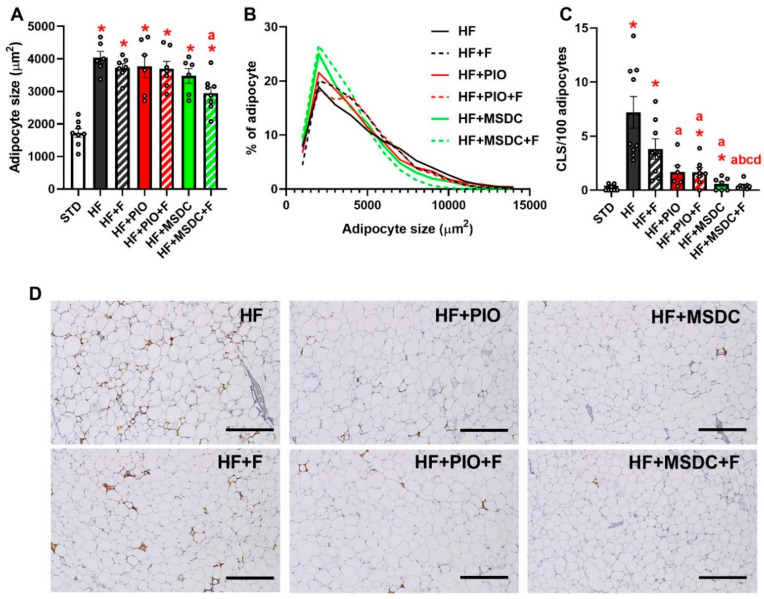
Morphology and immunohistochemistry of epididymal white adipose tissue (eWAT). Mice were fed STD or various HF diets for 8 weeks. Size of eWAT adipocytes was evaluated from hematoxylin + eosin stained sections (**A**) and expressed as mean (**A**) or histogram of adipocyte size ((**B**) pooled data from 4800 adipocytes). Inflammation of eWAT was assessed using the anti-Mac-2 antibody, which labeled macrophages aggregated around dying adipocytes ((**D**) reddish color; representative histological sections are shown; scale bar = 200 µm), and was expressed as the percentage of CLS/100 adipocytes (**C**). Data are means ± SEMs (*n* = 8). * Significantly different from STD (*p* ≤ 0.05, *t*-test). a—significantly different vs. HF; b—significantly different vs. HF + F; c—significantly different vs. HF + PIO; d—significantly different vs. HF + PIO + F (*p* ≤ 0.05, one-way ANOVA). For designation of dietary groups, see Section 2.1.

Suppression of the obesogenic effect of HF diet was mirrored by changes in weight of eWAT (elevated in response to HF diet), which was ≈2.1-fold lower in the HF + MSDC + F group compared to the HF group. Similar changes in adiposity were observed at the level of scWAT, but differences between the diets were less pronounced (Table 2). Histological and immunohistochemical analysis of eWAT revealed adipocyte hypertrophy induced by HF diet, which was significantly prevented only by HF + MSDC + F (Figure 2A,B). HF diet also induced low-grade eWAT inflammation, marked by CLS that are formed by macrophages aggregated around dying adipocytes [45]. This infiltration was completely counteracted by HF + MSDC + F and markedly reduced by all other interventions except HF + F (Figure 2C,D).

Liver weight was similar in the STD, HF and HF + PIO mice, but it was reduced (≈1.1 to ≈1.3-fold) in response to all the other interventions. Liver TAG content was ≈2.8-fold higher in the HF mice compared to the STD mice. HF + PIO tended to increase liver TAG content vs. HF, whereas it was reduced by both HF + F and HF + MSDC compared to HF + PIO. It was also decreased by both combined interventions compared with the HF diet and HF + PIO. The effect of HF + MSDC + F tended to be the most pronounced (≈2.2 and ≈1.6-fold decrease compared to the HF and HF + MSDC mice, respectively; Table 2).

### 3.2. Lipid and Glucose Homeostasis

Levels of plasma lipids, i.e., NEFA, TAG and cholesterol, were increased by HF diet compared to STD. All the interventions decreased plasma levels of these analytes, with the most pronounced effects being observed in the HF + MSDC + F mice. In these mice, the levels of all these lipid metabolites tended to be lower as compared with the HF + PIO + F mice (Table 2).

**Figure 3:**
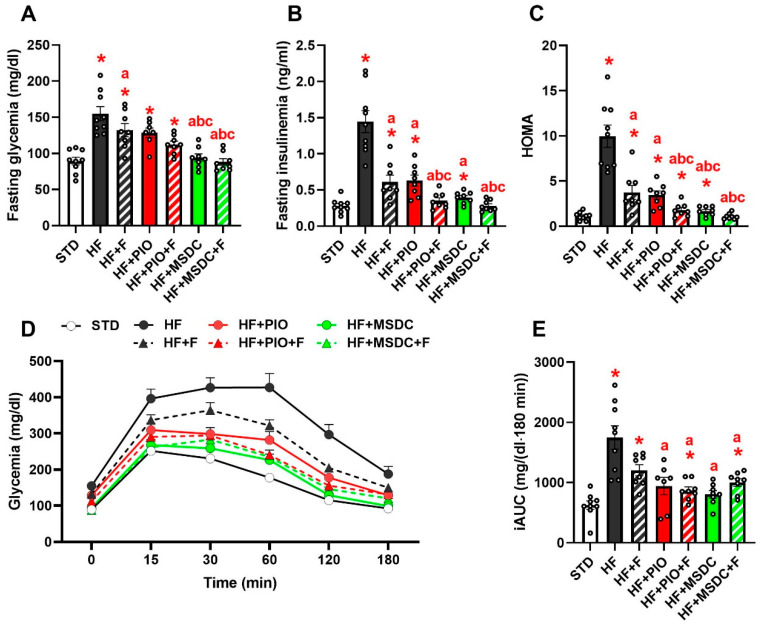
Glucose homeostasis. Mice were fed STD or various HF diets for 8 weeks. Glycemia (**A**) and insulinemia (**B**) after overnight fasting; HOMA index (**C**); intraperitoneal GTT: time course of glycemia during the test (**D**) and the corresponding incremental area under the curve (iAUC; (**E**)). Data are means ± SEMs (*n* = 8). * Significantly different from STD (*p* ≤ 0.05, *t*-test). a—significantly different vs. HF; b—significantly different vs. HF + F; c—significantly different vs. HF + PIO (*p* ≤ 0.05, one-way ANOVA). HOMA: homeostatic model assessment; GTT: glucose tolerance test. For designation of dietary groups, see Section 2.1.

HF diet increased both fasting blood glucose and plasma insulin levels. These effects were completely counteracted by both combined interventions (Figure 3A,B). The remaining interventions showed similar, though less pronounced influences. Changes in HOMA index indicated a complete normalization of glucose homeostasis by HF + MSDC + F and significant improvements by the remaining interventions (Figure 3C). Furthermore, the deterioration of glucose clearance during intraperitoneal GTT observed in the HF mice was ameliorated by all the interventions (except for HF + F), which all exerted similar effects (Figure 3D,E).

### 3.3. In Vivo Evaluation of TAG/FA Cycling Activity in eWAT

Next, we focused on the main goal of this study, verification of the hypothesis that the combined interventions using TZDs and Omega-3 exert an additive effect on TAG/FA cycling in WAT of mice fed an obesogenic HF diet. Therefore, this biochemical activity was characterized in eWAT, a typical WAT depot, using ^2^H_2_O administration in vivo and subsequent analysis of ^2^H enrichment of TAG.

**Figure 4:**
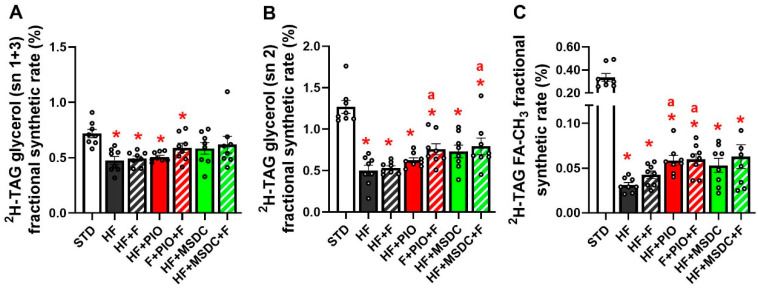
TAG synthesis and DNL in eWAT in vivo. Measurement of TAG synthesis (**A**,**B**) and DNL (FA de novo synthesis; (**C**)) in eWAT was performed using in vivo ^2^H_2_O incorporation in TAG in mice fed STD or various HF diets for 8 weeks. Analysis of total ^2^H enrichment in sn 1 + 3 (**A**) and sn 2 (**B**) position of glycerol, and in FA methyl moieties of TAG (**C**), was performed using NMR spectroscopy. Data are means ± SEMs (*n* = 8). * Significantly different from STD (*p* ≤ 0.05, *t*-test). a—significantly different vs. HF (*p* ≤ 0.05, one-way ANOVA). TAG: triacylglycerol; DNL: de novo lipogenesis; NMR: nuclear magnetic resonance. For designation of dietary groups, see Section 2.1.

Analysis of deuterated TAG using NMR spectroscopy enabled for separate evaluations of total enrichment of ^2^H in glycerol and FA methyl moieties of TAG, reflecting the rates of TAG glycerol 3-phosphate precursor synthesis (Figure 4A,B) and DNL activity (Figure 4C), respectively. The ^2^H enrichment of the glycerol moiety was evaluated separately for sn 1 + 3 (Figure 4A) and sn 2 positions (Figure 4B). The enrichment at all these positions was suppressed by HF diet compared with STD, ≈1.6 and ≈2.6-fold, respectively (Figure 4A,B). There was no significant effect of any intervention (i.e., difference between the HF-based diets) on sn 1 + 3 position if evaluated by 1-way ANOVA (Figure 4A), but there was a significant effect of TZDs if evaluated by 2-way ANOVA (one factor—Omega-3; another factor—TZD). The ^2^H enrichment at sn 2 position was affected more strongly: HF + F, HF + PIO and HF + MSDC tended to increase the enrichment, while both types of the combined intervention, i.e., HF + PIO + F and HF+ MSDC + F, exerted a significant stimulatory effect compared with the HF mice (≈ 1.5 and ≈1.6-fold, respectively) resulting in a substantial rescue of the fractional rate of TAG synthesis (Figure 4B).

The ^2^H enrichment of FA methyl moieties of TAG (DNL activity) was suppressed ≈10.8-fold by HF diet compared to STD (Figure 4B). This decrease tended to be counteracted by HF + F, HF + MSDC and HF + MSDC + F, whereas HF + PIO and HF + PIO + F exerted a significant stimulatory effect compared with the HF mice (≈1.9-fold). However, even in this case, DNL activity remained much lower as compared with the STD mice (Figure 4B).

**Figure 5:**
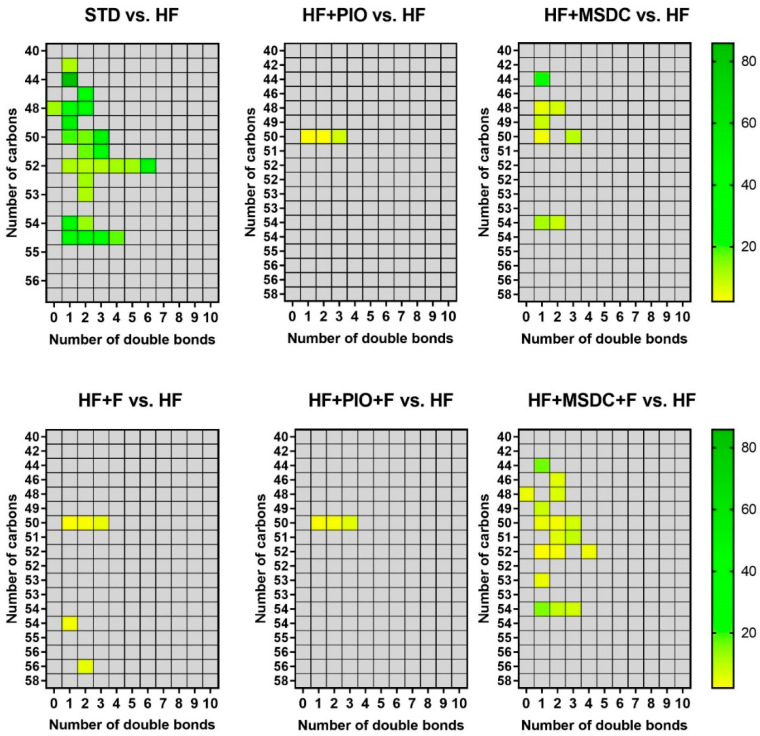
^2^H enrichment of various TAG species. In total, 62 deuterated TAG species detected using LC–MS analysis were considered. The ratio between mean enrichment of TAG in the respective intervention group and the HF mice (see the colored bar) was plotted only if the difference between groups was significant (*p* ≤ 0.05, *t*-test). Data are plotted according to the length and saturation of TAG. Data are means ± SEMs (*n* = 8). For designation of dietary groups, see Section 2.1.

Using LC–MS, in total 62 deuterated TAG species could be unequivocally detected in eWAT (Figure 5). Compared with the HF mice, their ^2^H enrichment differed between the intervention groups. The number of differentially enriched TAG species increased in the following order: HF + PIO < HF + PIO + F < HF + F < HF + MSDC < HF + MSDC + F < STD. These data documented the most pronounced remodeling of TAG in response to the combined intervention using Omega-3 and MSDC-0602K. The above data suggest additive stimulation of TAG/FA cycling in response to both combined interventions, with a more pronounced effect of HF + MSDC + F.

The LC–MS approach allowed for characterization of ^2^H enrichment of the individual FA moieties in TAG (Supplementary Table S1). Compared to STD mice, the enrichment of all eight FAs considered was several times lower in mice fed HF-based diets. When comparing various interventions in mice fed HF-based diets, only HF + MSDC + F exhibited a significant effect with FA 16:0 (palmitate; ≈2.0-fold increase vs. HF, with HF + PIO + F exhibiting almost the same effect). Additionally, with FA 18:0 (oleate), HF + MSDC + F exhibited a nearly significant stimulatory effect. These data are in agreement with the relatively strong effects of both combined interventions on TAG/FA cycling in eWAT.

**Figure 6:**
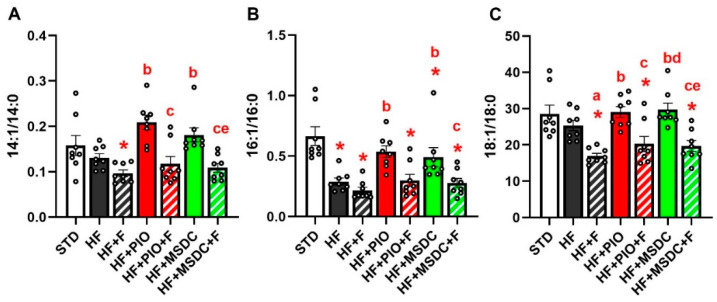
FA desaturation index in TAG. The ratios of the product of the desaturation and of the parent FA were calculated for myristic vs. myristoleic acid (14:1/14:0; **A**), palmitic vs. palmitoleic acid (16:1/16:0; **B**) and stearic vs. oleic acid (18:1/18:0; **C**). Data are means ± SEMs (*n* = 8). * Significantly different from STD (*p* ≤ 0.05, *t*-test). a—significantly different vs. HF; b—significantly different vs. HF + F; c—significantly different vs. HF + PIO; d—significantly different vs. HF + PIO + F; e—significantly different vs. HF + MSDC (*p* ≤ 0.05, one-way ANOVA). For designation of dietary groups, see Section 2.1.

Eventually, peak heights of selected deuterated monosaturated to saturated FA (i.e., FA desaturation index) were evaluated as a proxy for the activity of stearoyl-CoA desaturase (**SCD**) in the tissue (Figure 6) [46,47]. In all these cases (FA 14:1/14:0, FA 16:1/16:0, FA 18:1/18:0), the index tended to be decreased by HF diet, especially when Omega-3 was admixed to HF diet, with a significant suppression by HF + F vs. HF diet in the case of 18:1/18:0 index. As a single intervention, both TZDs neutralized the effect of HF diet. However, this positive effect of TZDs was prevented in the presence of Omega-3 in the diet. These results are consistent with the stimulation of DNL by both TZDs tested, and inhibition of DNL of some FA species by Omega-3 (see Discussion).

### 3.4. Gene Expression in Adipose Tissue

**Figure 7:**
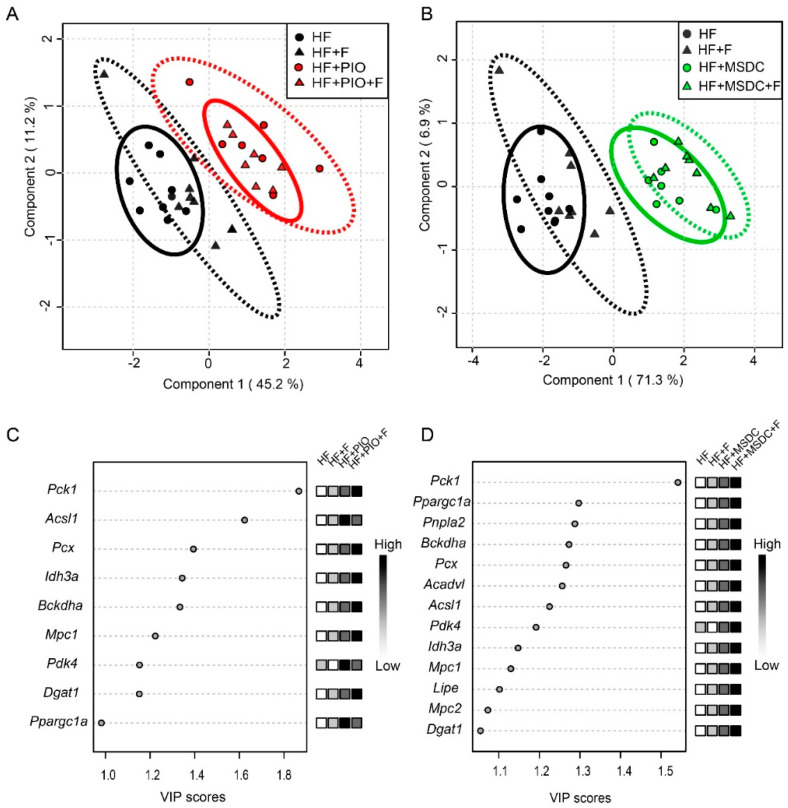
Multivariate analysis of gene expression in eWAT. Mice were fed various HF diets for 8 weeks. Expression of a total of 24 selected genes in eWAT was analyzed using PLS-DA (only genes with the expression level above background in all the groups were considered—i.e., all the genes in Figure 8, except for *Ucp1.* Score plots resulting from the analysis focus on the separation of the HF, HF + F, HF + PIO and HF + PIO + F mice (**A**), and the HF, HF + F, HF + MSDC and HF + MSDC + F mice (**B**). The corresponding variable importance in projection (VIP) plot with scores for identifying the most discriminating transcripts, with VIP > 1.0, is shown in (**C**) for (**A**), and in (**D**) for (**B**). The colored boxes indicate the relative expressions of the genes in each group. For designation of dietary groups, see Section 2.1.

Next, expression of 25 selected gene markers of regulatory pathways and metabolism in eWAT (reviewed in [2]) was evaluated using RT-qPCR. First, PLS-DA, a supervised classification method, was performed to obtain a global view on the effects of the interventions. In order to unmask possible differential effects of pioglitazone and MSDC-0602K (i) the STD mice were not considered, and (ii) the analysis was performed using two different subsets of mice. The analysis including the HF, HF + F, HF + PIO and HF + PIO + F mice (Figure 7A) revealed a separation within component 1, which described 45% of the total variation between the HF mice and two groups of mice fed PIO-containing diets (HF + PIO and HF + PIO + F), whereas Omega-3 had no effect. Similarly, the analysis focused on the potency of MSDC-0602K to modulate eWAT gene expression (Figure 7B) revealed a strong separation within component 1, which described 71% of the total variation between the HF mice and mice fed the two MSDC-containing diets (HF + MSDC and HF + MSDC + F), and a weak global effect of Omega-3 (HF vs. HF + F, and HF + MSDC vs. HF + MSDC + F, respectively). Therefore, both TZDs, pioglitazone and MSDC-0602K, exerted fundamental effects on eWAT gene expression, whereas the effect of MSDC-0602K was even more pronounced (compare Figure 7A,B). Moreover, this global analysis failed to reveal the major effect of Omega-3 on eWAT gene expression. Variable importance in projection (VIP) indicated that expression of the gene for PEPCK (*Pck1*) was the most discriminative factor within both types of analyses (compare Figure 7C,D).

**Figure 8:**
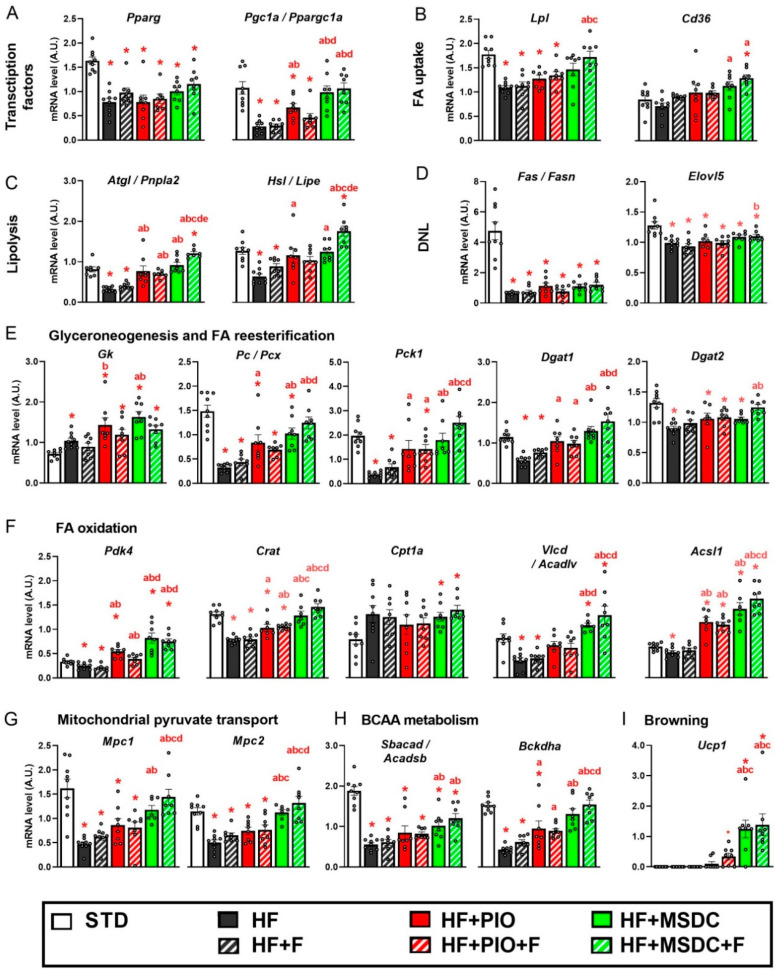
Expression of selected genes in eWAT. Mice were fed STD or various HF diets for 8 weeks. Expressions of the genes engaged in various regulatory and metabolic pathways (**A**–**I**) were evaluated using RT-qPCR and normalized to the mean signal of four reference genes (see Materials and Methods). Data are means ± SEMs (*n* = 8). * Significantly different from STD (*p* ≤ 0.05, *t*-test). a—significantly different vs. HF; b—significantly different vs. HF + F; c—significantly different vs. HF + PIO; d—significantly different vs. HF + PIO + F; e—significantly different vs. HF + MSDC (*p* ≤ 0.05, one-way ANOVA. For designation of dietary groups, see Section 2.1.

Next, expression of the gene markers (reviewed in [2,3]) was analyzed in eWAT in detail (Figure 8A–H). Expression of the gene encoding PPARγ (*Pparg*; for the abbreviations and gene names, see Table 1), the key transcription factor promoting differentiation of adipocytes and regulating their lipid metabolism, was downregulated by HF diet, and it was not affected by any intervention. The expression of the mitochondrial biogenesis-inducing PGC-1α gene (*Pgc1a/Ppargc1a*), which is a target of PPARγ was also reduced by HF diet. However, it was upregulated by HF + PIO, and even more by both HF + MSDC and HF + MSDC + F (Figure 8A).

Regarding FA uptake to WAT cells, expression of the genes for LPL but not CD36 (*Lpl* and *Cd36*, respectively) was downregulated by HF diet. Expression of both genes was elevated by HF + MSDC + F, whereas it remained unaffected by the other interventions (except for *Cd36* in the HF + MSDC mice; Figure 8B).

Expression of the genes of the key lipases ATGL and HSL (*Atgl/Pnpla2* and *Hsl/Lipe*, respectively) was downregulated by HF diet, and upregulated by most of the interventions, with the additive effect of HF + MSDC + F but not HF + PIO + F resulting in the former case in a higher expression than in the STD mice (Figure 8C).

Expression of the genes involved in FA synthesis (i.e., DNL), namely, FA synthase (*Fas/Fasn*) and FA elongase 5 (*Elovl5*), was strongly downregulated by HF diet, and it was not affected by any intervention (vs. HF diet; Figure 8D).

Among the markers of glyceroneogenesis and FA re-esterification (Figure 8E), expression of the gene for GK (*Gk*) was upregulated by HF diet, and it was also increased by both TZDs, but not by Omega-3. Expression of the genes engaged in glyceroneogenesis in WAT, namely, PC (*Pc/Pcx*) and PEPCK (*Pck1*), was strongly downregulated by HF and upregulated by both TZDs. The stimulatory effect of MSDC-0602K was more pronounced, with the combined HF + MSDC + F (but not HF + PIO + F) intervention showing an additive effect. Expression of the genes for DGAT1 and DGAT2 (*Dgat1* and *Dgat2*, respectively), involved in FA re-esterification [48], was suppressed by HF diet. Expression of *Dgat1* was upregulated by all the interventions, except for HF + F, with the combined HF + MSDC + F (but not HF + PIO + F) intervention showing an additive effect. Expression of *Dgat2* was only affected by the HF + MSDC + F, which normalized the expression to the level in STD group.

Regarding FA oxidation (Figure 8F), expression of the gene for PDK4 (*Pdk4*), which limits glucose oxidation by inhibiting pyruvate dehydrogenase and thus supports β-oxidation [49], was downregulated by HF diet. It was increased by both TZDs, with a stronger effect of MSDC-0602K, and it was insensitive to Omega-3. A very similar pattern was observed with the gene for CRAT (*Crat*), which acts in concert with PDK4 to regulate mitochondrial fuel oxidation, and which is associated with insulin sensitivity [50]. Expression of CPT1α gene (*Cpt1a*), encoding protein essential for the transport of FAs for their β-oxidation in mitochondria, was upregulated by HF diet, and the expression was similar across all the interventions. Expression of gene for VLCAD (*Vlcad/Acadvl*), enzyme catalyzing the first step of mitochondrial β-oxidation was downregulated by the HF diet, and it was upregulated by the two interventions containing MSDC-0602K. *Vlcad/Acadvl* gene expression in both HF + MSDC and HF + MSDC + F was even higher compared to the STD mice. However, *Vlcad/Acadvl* expression was unaffected by pioglitazone. The expression of gene for ACSL1 (*Acsl1*), which converts free long-chain FA into fatty acyl-CoA esters, was only marginally downregulated by the HF diet; it was not affected by HF + F. It was strongly upregulated by both TZDs, with the most pronounced effect being in HF + MSDC + F mice.

Expression of the genes for MPC1 and MPC2 (Figure 8G), the two forms of mitochondrial pyruvate carrier (*Mpc1* and *Mpc2*; [51]), was downregulated by HF diet. It was induced by MSDC-0602K (but not pioglitazone), independently of Omega-3.

The gene screen data above document a general decrease in the metabolic activities of eWAT in response to HF diet. This was apparent across the gene markers of all the metabolic pathways studied (except for *Gk*, *Cd36* and *Cpt1a*). Although both tested TZDs ameliorated the inhibitory effect of HF diet on the expression of most genes, MSDC-0602K exerted a stronger effect. Both TZDs promoted lipolysis and FA re-esterification, in association with the elevation of mitochondrial β-oxidation. The induction of FA oxidation was documented using the measurements of ^14^C-palmitate oxidation in fragments of eWAT dissected from the mice at the end of the dietary interventions (Supplementary Figure S1). It was in concert with the induction of mitochondrial biogenesis, documented by the increase in both the *Pgc1a/Ppargc1a* expression and the specific content of the complex III of mitochondrial respiratory chain in eWAT (Supplementary Figure S2). FA re-esterification was supported by enhanced activity of glyceroneogenesis mediated by PC and PEPCK, one of the most affected pathways by the studied interventions, but not by DNL. This indicated that induction of glycerol 3-phosphate by various interventions (see above) resulted mainly from glyceroneogenesis (see Discussion).

In general, Omega-3 tended to augment the stimulatory effect of MSDC-0602K, and this combination even exerted a significant additive effect on lipolysis-related genes (*Atgl/Pnpla2* and *Hsl/Lipe*). In contrast, Omega-3 tended to counteract the stimulatory effects of pioglitazone, with a significant influence at the level of PDK4. Pyruvate transport to mitochondria could be induced by MSDC-0602K, but not by pioglitazone or Omega-3.

Next, we focused on the expression of the genes for enzymes engaged in metabolism of branched-chain amino acids (BCAA), due to the role of BCAA metabolism in WAT in whole-body insulin sensitivity [52,53] and its improvement by TZDs [54]. Expression of these genes, namely, *Sbacad/Acadsb* and *Bckdha*, was downregulated by HF diet (Figure 8H). While pioglitazone increased the expression of *Bckdha*, MSDC-0602K stimulated the expression of both genes more than pioglitazone. Even in this case, Omega-3 supplementation (i.e., the HF + F diet) had no significant effect, but it tended to augment the stimulatory effect of MSDC-0602K. These data are consistent with the involvement of BCAA metabolism in WAT of diet-induced obese mice in the prevention of insulin resistance by both TZDs.

Lastly, expression of the gene for UCP1, which mediates thermogenesis in both brown and brite adipocytes (*Ucp1*; reviewed in [1]), was evaluated (Figure 8I). No expression above the background could be observed in the STD, HF or HF + F mice. In the remaining groups, *Ucp1* was expression was induced to various extents, with both MSDC-0602K-containing diets exerting an equal effect, higher compared with the other diets.

**Figure 9:**
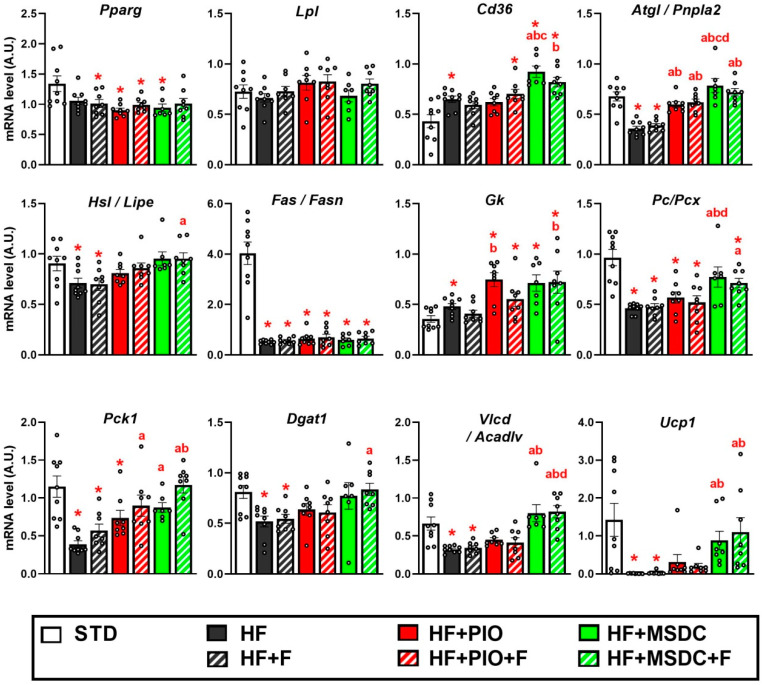
Expression of selected genes in subcutaneous WAT. Mice were fed STD or various HF diets for 8 weeks. Expression of the subset of genes shown in Figure 8 was assessed using RT-qPCR and normalized to the mean signal of four reference genes (see Materials and Methods). Data are means ± SEMs (*n* = 8). * significantly different from STD (*p* ≤ 0.05, *t*-test). a—significantly different vs. HF; b—significantly different vs. HF + F; c—significantly different vs. HF + PIO; d—significantly different vs. HF + PIO + F. For designation of dietary groups, see Section 2.1.

In order to learn whether the above described effects of various dietary interventions on gene expression in eWAT depended on the anatomical location of the tissue, expression levels of several of the studied genes were also evaluated in scWAT of the same animals (Figure 9). The gene expression pattern was very similar in both WAT depots compared. Only *Ucp1* expression showed fat depot-specific differences, namely, a remarkably scattered expression in scWAT of the STD-fed mice (Figure 9), which contrasted with the lack of *Ucp1* expression in eWAT of these animals (Figure 8I). Feeding HF diet eliminated *Ucp1* expression in scWAT, while the two MSDC-0602K-containing diets rescued the expression to the levels observed in the STD mice (Figure 8I). When the relative levels of *Ucp1* expression were compared across eWAT, scWAT and interscapular brown fat, the highest levels found in the two WAT depots were not significantly different, and they were two orders of magnitude lower compared with those in brown adipose tissue of these mice (not shown).

## 4. Discussion

**Figure 10:**
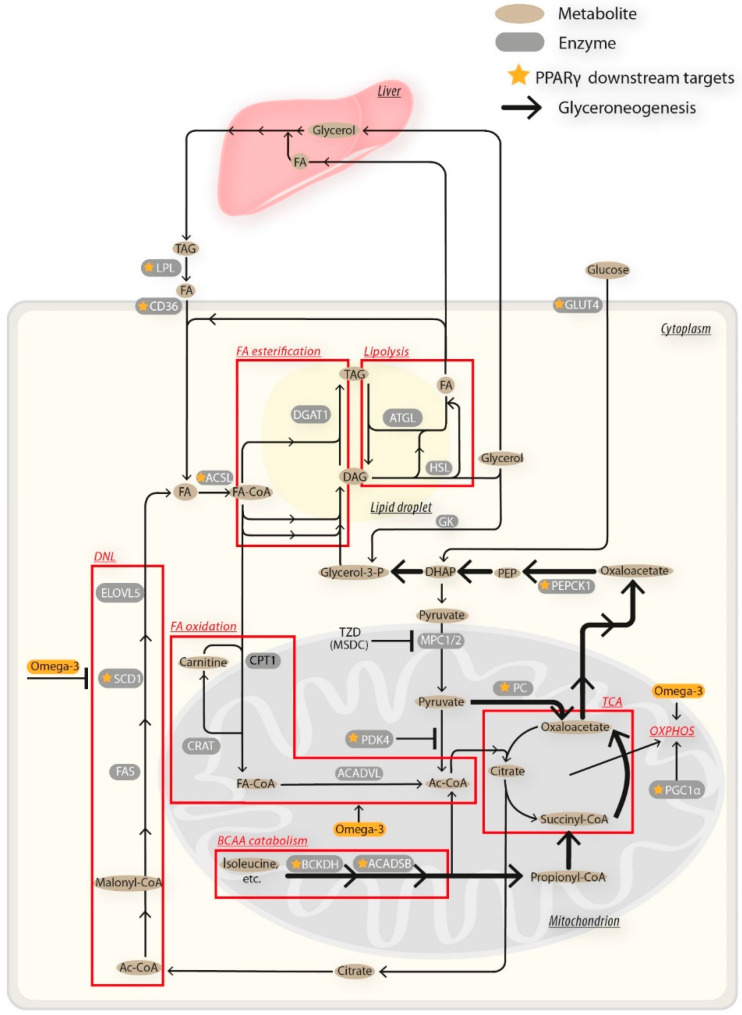
Hypothetical model for the complex mechanism of the induction of TAG/FA cycling activity in WAT by the combined intervention using Omega-3 and TZDs in adipocytes. Both pioglitazone and MSDC-0602K stimulate transcriptional factor PPARγ and its targets, resulting in increased glucose and FA uptake into the adipocyte. In parallel, mitochondrial FA oxidation is upregulated compared with glucose oxidation, reflecting (i) higher FA influx; (ii) higher Omega-3 mediated induction of genes involved in FA oxidation, mitochondrial biogenesis and OXPHOS activity; (iii) inhibition of pyruvate transport to mitochondria—especially in response to MSDC-0602K; and (iv) induction of PDK4, which results in inhibition of oxidation of a limited amount of pyruvate entering mitochondria. Thus, synthesis of glycerol-3-phosphate (glycerol-3-P) is increased by both conversion from glucose and glyceroneogenesis from pyruvate. Induction of BCAA catabolism provides another source of glyceroneogenic substrate oxaloacetate. The increased levels of glycerol-3-P and FA-CoA stimulate synthesis of TAG, linked to concomitant increase in lipolysis. FA released from TAG can be either oxidized or re-esterified (in TAG/FA cycle). The re-esterification can occur in adipocytes (primary FA re-esterification) or extra-adipose tissues (secondary FA re-esterification), mainly the liver (reviewed in [29,32]). Glycerol released during lipolysis is transported via blood to the liver to support (i) gluconeogenesis, and (ii) the formation of glycerol-3-P for TAG synthesis, either from glucose, or via direct phosphorylation of glycerol. For other details, references and abbreviations, see the main text and Table 1. For the explanation of the pleiotropic effects of TZDs, see also [22]. Ac—acetyl; CoA—coenzyme A; G3P—glyceraldehyde-3-phosphate; LPA—lysophosphatidic acid; P—phosphate.

The principal finding of this report was that additive beneficial effects of Omega-3 and TZDs in mice fed obesogenic HF diet were associated with pronounced changes in gene expression (Figure 8) and metabolism of eWAT. These effects included the reduction in body weight gain and adiposity (Figure 1); preservation of glucose homeostasis and dyslipidemia (Figure 3 and Table 2); and reduction in liver fat content (Table 2) and low-grade inflammation of WAT (Figure 2C). In terms of WAT function, the results suggest the role of TAG/FA cycling (Figure 10) in the beneficial effects of the studied interventions.

To our knowledge, this is the first study to characterize the direct effects of the combined intervention using Omega-3 and TZDs on WAT metabolism. Our results document a decrease of metabolic activities in eWAT by HF diet [55], and a partial counteraction of this effect by various interventions. Thus, the measurements of glyceroneogenesis using in vivo labeling of TAG by deuterium from ^2^H_2_O, and subsequent analyses of TAG composition using NMR spectroscopy and LC–MS, documented an additive interaction between Omega-3 and both types of TZDs in the stimulation of this activity. FA re-esterification was stimulated the most by HF + MSDC + F, as documented by the highest number of deuterated TAG species (detected using LC–MS) in response to this intervention vs. HF diet. Additive stimulation by Omega-3 and TZDs, with a stronger effect of HF + MSDC + F, was observed in both eWAT and scWAT, as documented by the expression of the genes involved in FA uptake (*Lpl* and *Cd36*), lipolysis (*Atgl/Pnpla2* and *Hsl/Lipe*), glyceroneogenesis (*Pc/Pcx* and *Pck1*), FA re-esterification (*Dgat1*) and FA oxidation (*Crat*, *Vlcd/Acadlv* and *Acsl1*), and the biochemical data. The changes in gene expression suggested that stimulation of glyceroneogenesis depended more on PC-PEPCK-mediated glycerol 3-phosphate formation rather than GK, which is also consistent with the preferential activation of the earlier pathway [29,38] and the induction of *Pdk4* [35] by TZD. However, the relatively low upregulation of *Gk* expression, which was also observed in both eWAT and scWAT in response to various interventions, suggested that direct phosphorylation of glycerol was also involved in the increased formation of glycerol 3-phosphate. Thus, the activation of TAG/FA cycling in response to various interventions could be even higher than illustrated by the measurements of glyceroneogenesis using labeling of TAG by deuterium from ^2^H_2_O (see above). That *Dgat1* but not *Dgat2* expression was affected reflects (i) the interaction of DGAT1 with ATGL during lipolysis and FA re-esterification, and (ii) the involvement of DGAT2 in the esterification of FA formed by DNL [48]. Collectively, the data document the additive stimulation of TAG/FA cycling by Omega-3 and TZDs, especially by the combined intervention that involved MSDC-0602K.

Direct measurement of DNL in vivo in eWAT and the corresponding gene expression analysis (*Fas/Fasn*) indicated very strong suppression of DNL by HF diet, and a limited rescue of this activity by both TZDs [56]. Moreover, the effect of HF diet was further augmented by Omega-3 [57]; these FAs even prevented activation of DNL by TZDs. These results were further supported by the changes in the FA desaturation index, marking SCD activity and lipogenesis in the tissue [46,47]. Interestingly, stimulation of TAG/FA cycling in WAT by prolonged cold exposure of mice was accompanied by a pronounced stimulation of DNL in the tissue [33], documenting independent regulation of glyceroneogenesis (TAG/FA cycling) and DNL in WAT.

Molecular mechanisms of the combined intervention on eWAT gene expression and metabolism probably involve interactions of the tested compounds with intracellular regulatory pathways in adipocytes, including PPARs, endocannabinoids, AMP-activated protein kinase and others (reviewed in [1,2,22,33]). These Omega-3-induced changes result, namely, in increased capacity for FA oxidation in the mitochondria, linked with OXPHOS (Figure 10). Some effects in WAT may be secondary to the activation of lipid metabolism in the liver and other tissues, especially due to the PPARα-mediated response to Omega-3 [58]. The effects of TZDs are even more pleiotropic, reflecting the differential activation of multiple PPARy targets, and the reversible inhibition of pyruvate transport into mitochondria ([25,26,27]; Figure 10). Despite the additive stimulation by Omega-3 and TZDs of most of the metabolic activities of WAT, which are the basis of the “healthy adipocyte” phenotype (reviewed in [1,2,3]), some metabolic pathways were affected differently. Thus, DNL is inhibited by Omega-3 [57] and stimulated by TZDs ([56]; see above), reflecting probably the differential control via SCD1 [47,59].

Moreover, stimulation of glucose uptake due to the insulin-sensitizing effect of TZDs probably plays a major role in the stimulation of DNL. Expression of the genes of BCAA metabolism was upregulated by TZDs [54], independent of Omega-3, in agreement with the role of BCAA in the insulin-sensitizing effect of TZDs [52,53]. Overall, the data suggest a complex modulation of WAT metabolic activities, which converge in the TAG/FA cycle. The most pronounced effects were produced by the combination of HF + MSDC + F. This may indicate that the modulation of pyruvate’s entry into mitochondria by MSDC-0602K has a major impact on these processes [25,26,27]. As shown graphically in Figure 10, the effects observed in WAT in this study involve mitochondrial and extramitochondrial metabolism in both the adipose tissue and the liver. The Omega-3 and TZD interactions can occur at various regulatory levels and may also involve both direct and downstream modulation of PPARγ, and likely other transcriptional networks (Figure 10).

Our results support the role of TAG/FA cycling in WAT in a healthy metabolic phenotype. This core biochemical activity of WAT is essential for flexible control of plasma NEFA levels and supply of FA to serve as energy fuel [1,29,30,31,32]. Insufficient capacity of TAG/FA cycling in WAT probably underlies insufficient “expandability” of WAT [60], i.e., pathological low capacity of this tissue to serve as a buffer for circulating FAs. This leads to lipotoxic damage to metabolism and insulin sensitivity in extra-adipose tissues. Indeed, in WAT of obese [61] and diabetic patients [62], the activities of both TAG/FA cycling and DNL are relatively low. Additionally, OXPHOS slows down in obesity and with aging [63], whereas it is increased by exercise [64]. Thus, the additive stimulatory effect of TZDs and Omega-3 on TAG/FA cycling in WAT could provide an important determinant of metabolic health.

The experimental design was based on the doses of Omega-3 and TZDs used in the previous studies on HF diet-fed mice (see Section 2.1). Regarding the TZDs, when re-calculated to human equivalent dose (HED; [65]), 50 mg pioglitazone/kg of the HF + PIO or HF + PIO + F diet was equivalent to 32 mg HED (with 15–45 mg pioglitazone/day, the usual therapeutic dose in diabetic patients), whereas 331 mg MSDC-0602K/kg of the HF + MSDC or HF + MSDC + F diet was equivalent to 146 mg HED (with 60–250 mg MSDC-0602K/day used in a clinical trial [28]). The ratio between the dose of MSDC-0602K and pioglitazone, i.e., ≈6.6, was relatively low with respect to the ≈16-fold lower affinity of MSDC-0602K to PPARγ as compared with pioglitazone [25]. Nevertheless, the effects of MSDC-0602K were mostly stronger compared with pioglitazone. These differences were especially pronounced in the combined interventions HF + MSDC + F and HF + PIO + F (see below). The relatively high potency of MSDC-0602K was following the notion that, unlike pioglitazone, the effects of MSDC-0602K depended largely on the direct inhibition of mitochondrial pyruvate carriers MPC1 and MPC2, rather than on the activation of PPARγ-mediated transcription [25,26,27]. Indeed, expression of *Mpc1* and *Mpc2*, which was downregulated by HF diet, was increased by HF + MSDC and HF + MSDC + F, but not by any other intervention. This suggests an adaptive response at the level of *Mpc1* and *Mpc2* transcription.

The effect of the interventions on gross energy balance (i.e., lower adiposity accretion in face of equal energy intake in all the HF-based diets compared) needs to be clarified, especially for diets containing MSDC-0602K. The present data could not rule out a possibility that a decrease in dietary energy intake in the gut was involved. Consistent with the previous studies (reviewed in [1]), we have observed a small increase in *Ucp1* expression in WAT, reaching much lower levels than in BAT. Thus, UCP1-mediated thermogenesis could be involved, but its characterization was out of the scope of this study.

Regarding the involvement of energy expenditure in the anti-obesity effects of the studied interventions, a seminal recent study should be recalled [31]. It highlighted the role of futile metabolic cycling of circulating metabolites for a steady supply of appropriate nutrients to each tissue, despite varying dietary intake. It demonstrated that (i) a majority of circulating carbon flux reflects glucose-lactate and TAG-glycerol-FA cycling, and (ii) lactate and FA are the two major energy fuels. The rate of this futile cycling was much faster [31] than so far assumed [29,32]. As shown before [32], the flux of circulating FA depends almost entirely on TAG/FA cycling in WAT. Thus, the energy requirements for TAG/FA cycling in WAT, associated with futile cycling of circulating FA and glycerol, must be relatively high. This energy expenditure should affect significantly whole-body energy balance. This could largely explain the anti-obesity effects of various interventions. Indeed, the HF + MSDC + F intervention that resulted in the highest stimulation of TAG turnover in eWAT exerted the most pronounced reduction of HF diet-induced obesity. Accordingly, as we have shown recently in the obesity-resistant A/J and obesity-prone B6 mice exposed to cold stress [33], obesity resistance is associated with higher stimulability of TAG/FA cycling in eWAT.

## 5. Conclusions

Modulation of intrinsic metabolic features of WAT, and in particular stimulating the level of TAG/FA cycling, is involved in additive beneficial effects of Omega-3 and TZDs on metabolic health in diet-induced obese mice. Whereas TAG/FA cycling in WAT is suppressed by obesogenic HF diet, its partial rescue linked to the increased flux of circulating metabolites could reduce obesity. WAT metabolism represents an important target for treatment strategies for obesity and associated diseases. Combined interventions using Omega-3 could both augment the positive effects of TZD insulin sensitizers and minimize/prevent the weight gain that can occur with this pharmacotherapy. The second-generation PPARγ-sparing TZDs represent prospective potent pharmaceuticals for use in the combined intervention.

## Figures and Tables

**Table 1:** Sequences of primers.

Gene Name	Gene ID	Forward Primer	Reverse Primer
*Acsl1*	14081	GAAGCCGTGGCCCAGGTGTTTGTC	TTCGCCTTCAGTGTTGGAGTCAGA
*Actb*	11461	GAACCCTAAGGCCAACCGTGAAAAGAT	ACCGCTCGTTGCCAATAGTGATG
*Atgl/Pnpla2*	66853	GGCAATCAGCAGGCAGGGTCTTTA	GCCAACGCCACTCACATCTACG
*Bckdha*	12039	ACGGCGGGCTGTGGCTGAGAA	GAGATTGGGTGGTCCTGCTTGTCC
*Cd36*	12491	TGATACTATGCCCGCCTCTCC	TTCCCACACTCCTTTCTCCTCTAC
*Cpt1a*	12894	GCAGCTCGCACATTACAAGGACAT	AGCCCCCGCCACAGGACACATAGT
*Crat*	12908	ACATGGTGGTGGTAGCAAGTTCAA	GGCAAGGGCACCATAGGAGA
*Cyphb/Ppib*	19035	GGGAGATGGCACAGGAGGAAAGAG	ACCCAGCCAGGCCCGTAGTG
*Dgat1*	13350	TGGCCAGGACAGGAGTATTTTTGA	CTCGGGCATCGTAGTTGAGCA
*Dgat2*	67800	TGCCCTACTCCAAGCCCATCACC	TCAGTTCACCTCCAGCACCTCAGTCTC
*Eef1a1*	13627	TGACAGCAAAAACGACCCACCAAT	GGGCCATCTTCCAGCTTCTTACCA
*Eef2*	13629	GAAACGCGCAGATGTCCAAAAGTC	GCCGGGCTGCAAGTCTAAGG
*Elovl5*	68801	CCTCTCGGGTGGCTGTTCTTCC	AGGCTTCGGCTCGGCTTGTC
*Fas/Fasn*	14104	TGGGTGTGGAAGTTCGTCAG	GTCGTGTCAGTAGCCGAGTC
*Gk*	14933	TCGTTCCAGCATTTTCAGGGTTAT	TCAGGCATGGAGGGTTTCACTACT
*Hsl/Lipe*	16890	TGCGCCCCACGGAGTCTATGC	CTCGGGGCTGTCTGAAGGCTCTGA
*Lpl*	16956	AGCCCCCAGTCGCCTTTCTCCT	TGCTTTGCTGGGGTTTTCTTCATTCA
*Mpc1*	55951	TCATTCAGGGAGGACGACTTATC	TGTTTTCCCTTCAGCACGACTAC
*Mpc2*	70456	CTCCCACCCTGCTGCTGTCG	GGCCTGCCGGGTGGTTGTA
*Pc/Pcx*	18563	CCCCTGGATAGCCTTAATACTCGT	TGGCCCTTCACATCCTTCAAA
*Pck1*	18534	GGCAGCATGGGGTGTTTGTAGGA	TTTGCCGAAGTTGTAGCCGAAGAAG
*Pdk4*	27273	GGCTTGCCAATTTCTCGTCTCTA	TTCGCCAGGTTCTTCGGTTCC
*Pgc1a/Ppargc1a*	19017	CCCAAAGGATGCGCTCTCGTT	TGCGGTGTCTGTAGTGGCTTGATT
*Pparg*	19016	GCCTTGCTGTGGGGATGTCTC	CTCGCCTTGGCTTTGGTCAG
*Sbacad/Acadsb*	66885	GCATCTGAGGTCGCTGGGCTAAC	CGATGTGCTTGGCGATGGTGT
*Ucp1*	22227	CACGGGGACCTACAATGCTTACAG	CACGGGGACCTACAATGCTTACAG
*Vlcad/Acadvl*	11370	CAGGGGTGGAGCGTGTGC	CATTGCCCAGCCCAGTGAGTTCC

Abbreviations: *Acsl1*, acyl-CoA synthetase long-chain family member 1 (ACSL1); *Actb*, actin beta (ACTB); *Atgl/Pnpla2*, adipose triglyceride lipase, also known as patatin-like phospholipase domain-containing protein 2 (ATGL); *Bckdha*, branched chain ketoacid dehydrogenase E1, alpha polypeptide (BCKDHA); *Cpt1a*, carnitine palmitoyltransferase 1a (CPT1A); *Crat*, carnitine acetyltransferase (CRAT); *Cyphb/Ppib*, cyclophilin beta, also known as peptidylprolyl isomerase B (CYPHB); *Dgat1*, diacylglycerol O-acyltransferase 1 (DGAT1); *Dgat2*, diacylglycerol O-acyltransferase 2 (DGAT2); *Eef1a1*, eukaryotic translation elongation factor 1 alpha 1 (EEF1A1); *Eef2*, eukaryotic translation elongation factor 2 (EEF2); *Elovl5*, ELOVL family member 5, elongation of long-chain fatty acids (ELOVL5); *Fas/Fasn*, fatty acid synthase (FAS); *Gk*, glycerol kinase (GK); *Hsl/Lipe*, lipase, hormone sensitive (HSL); *Lpl*, lipoprotein lipase (LPL); *Mpc1*, mitochondrial pyruvate carrier 1 (MPC1); *Mpc2*, mitochondrial pyruvate carrier 2 (MPC2); *Pc/Pcx*, pyruvate carboxylase (PC); *Pck1*, phosphoenolpyruvate carboxykinase 1, cytosolic (PCK1); *Pdk4*, pyruvate dehydrogenase kinase, isoenzyme 4 (PDK4); *Pgc1a/ Ppargc1a*, peroxisome proliferative activated receptor gamma, coactivator 1 alpha (PGC1A); *Pparg* peroxisome proliferator activated receptor gamma (PPARG); *Sbacad/Acadsb*, acyl-Coenzyme A dehydrogenase, short/branched chain (SBACAD); *Ucp1*, uncoupling protein 1 (UCP1); *Vlcad/Acadvl*, acyl-Coenzyme A dehydrogenase, very long chain (VLCAD).

**Table 2:** Body mass, tissue weights, tissue TAG content and plasma parameters in mice fed various diets.

	STD	HF	HF + F	HF + PIO	HF + PIO + F	HF + MSDC	HF + MSDC + F
Energy balance							
Body weight initial g)	27.2 ± 0.40	27.6 ± 0.40	27.9 ± 0.49	27.3 ± 0.47	27.9 ± 0.48	27.8 ± 0.42	27.8 ± 0.50
Body weight final (g)	32.9 ± 0.70	44.3 ± 0.64 *	41.0 ± 1.13 *	41.5 ± 1.42 *	38.6 ± 1.35 *^,a^	36.9 ± 1.16 *^,a^	34.5 ± 0.91 ^abc^
Body weight gain (g)	5.50 ± 0.44	16.1 ± 0.63 *	12.0 ± 0.75 *^,a^	13.3 ± 1.17 *	10.1 ± 1.11 *^,a^	8.27 ± 0.94 ^abc^	6.02 ± 0.58 ^abcd^
Cumulative food intake (MJ/animal)	3.86 ± 0.06	4.59 ± 0.11 *	4.21 ± 0.18	4.57 ± 0.25 *	4.17 ± 0.15	4.75 ± 0.20 *	4.81 ± 0.32 *
Tissues							
eWAT weight (mg)	833 ± 73	2489 ± 84 *	1977 ± 88 *^,a^	2252 ± 168 *	1734 ± 142 *^,ac^	1511 ± 123 *^,ac^	1171 ± 117 *^,abcd^
scWAT weight (mg)	403 ± 34	1398 ± 87 *	1283 ± 137 *	1614 ± 288 *	1191 ± 84 *	1097 ± 96 *	936 ± 78 *
Liver weight (mg)	1763 ± 43	1793 ± 63	1563 ± 58 *^,a^	1613 ± 84 *	1477 ± 58 *^,a^	1425 ± 58 *^,a^	1418 ± 33 *^,a^
Liver TAG content (mg/g)	33.2 ± 1.16	93.4 ± 8.03 *	57.0 ± 4.96 *^,a^	112 ± 17.6 *^,b^	48.6 ± 5.77 *^,ac^	67.9 ± 10.0 *^c^	42.4 ± 5.58 ^ac^
Plasma (random fed state)							
NEFA (mmol/L)	0.50 ± 0.05	0.66 ± 0.04 *	0.38 ± 0.03 *^,a^	0.42 ± 0.02 ^a^	0.34 ± 0.03 *^,a^	0.38 ± 0.04 *^,a^	0.22 ± 0.02 *^,abce^
TAG (mmol/L)	1.39 ± 0.06	1.79 ± 0.05 *	1.05 ± 0.09 *^,a^	0.99 ± 0.06 *^,a^	0.82 ± 0.06 *^,a^	0.89 ± 0.06 *^,a^	0.54 ± 0.04 *^,abce^
Cholesterol (mmol/L)	2.81 ± 0.33	4.61 ± 0.14 *	3.40 ± 0.12 ^a^	3.91 ± 0.10 *^,ab^	2.91 ± 0.13 ^ac^	3.40 ± 0.13 ^ac^	2.55 ± 0.07 ^abce^

Data are means ± SEMs (*n* = 16–19). Cumulative energy intake was assessed during the initial 7-week period of dietary interventions. * Significantly different from STD (*p* ≤ 0.05, t-test). ^a^ Significantly different vs. HF; ^b^ significantly different vs. HF + F; ^c^ significantly different vs. HF + PIO; ^d^ significantly different vs. HF + PIO + F; ^e^ significantly different vs. HF + MSDC (*p* ≤ 0.05, one-way ANOVA). STD: standard chow diet; HF: high-fat; PIO: pioglitazone; eWAT: epididymal white adipose tissue; scWAT: subcutaneous white adipose tissue; TAG: triacylglycerol; NEFA, non-esterified fatty acid. For designation of dietary groups, see Section 2.1.

## Supplementary Materials

The following are available online at https://www.mdpi.com/2072-6643/12/12/3737/s1. Method:LC–MS-based lipidomics. Figure S1: Palmitate oxidation in eWAT. Figure S2: Quantification of the complexes of mitochondrial respiratory chain in eWAT. Table S1: ^2^H enrichment of various FA moieties in response to the interventions.
