# Supplementary material for: Additive Effects of Omega-3 Fatty Acids and Thiazolidinediones in Mice Fed a High-Fat Diet: Triacylglycerol/Fatty Acid Cycling in Adipose Tissue
Source: Nutrients. 2020 Dec 4;12(12):3737. doi: 10.3390/nu12123737 (PMC7761951; doi:10.3390/nu12123737)
Supplement: Supplementary file 1 [file nutrients-12-03737-s001.pdf]

Supplementary Materials

Materials and Methods

LC-MS-based lipidomics

White adipose tissue (**WAT**) samples (20 mg) were homogenized with 275 μL of methanol and 275 μL of 10% methanol for 1.5 min using a grinder (MM400, Retsch, Germany). Then, 1 mL of methyl *tert*-butyl ether (**MTBE**) was added, the tubes were shaken for 1 min, and centrifuged at 16,000 rpm for 5 min.

For profiling of high-abundant triacylglycerols (**TAG**), 10 μL of the upper organic phase was collected, diluted with 990 μL of methanol containing CUDA internal standard, shaken for 30 s, centrifuged at 16,000 rpm for 2 min, and used for the lipidomics ESI(+) platform.

For profiling of free fatty acids after hydrolysis, 50 μL of the upper organic phase was collected into a 2 mL tube with a screw cap, evaporated, dissolved in 500 μL of 0.3 M KOH in a methanol:water mixture (9:1, *v*/*v*), and shaken at 55°C for 1 hour at 200 rpm. Then, 60 μL of 3 M HCl in methanol was added (pH ~6), followed by 500 μL of water and 500 μL of hexane. The mixture was shaken for 30 s and centrifuge at 16,000 rpm for 5 min. A volume of 200 μL of the upper phase (hexane) was evaporated and dissolved in 200 μL of a dichloromethane:methanol:isopropanol mixture (1:2:4, *v*/*v*/*v*), shaken for 30 s, and centrifuge at 16,000 rpm for 5 min. After dilution (1:10) with methanol containing CUDA internal standard, the extracts were used for the lipidomics ESI(−) platform.The LC-MS system consisted of a Vanquish UHPLC System (Thermo Fisher Scientific, Bremen, Germany) coupled to a Q Exactive Plus mass spectrometer (Thermo Fisher Scientific, Bremen, Germany).

Lipids were separated on an Acquity UPLC BEH C18 column (50 × 2.1 mm; 1.7 μm) coupled to an Acquity UPLC BEH C18 VanGuard pre-column (5 × 2.1 mm; 1.7 μm) (Waters, Milford, MA, USA). The column was maintained at 65°C at a flow-rate of 0.6 mL/min. For LC–ESI(+)-MS analysis, the mobile phase consisted of (A) 60:40 (*v*/*v*) acetonitrile:water with ammonium formate (10 mM) and formic acid (0.1%) and (B) 90:10:0.1 (*v*/*v*/*v*) isopropanol:acetonitrile:water with ammonium formate (10 mM) and formic acid (0.1%). For LC–ESI(−)-MS analysis, the composition of the solvent mixtures were the same with the exception of the addition of ammonium acetate (10 mM) and acetic acid (0.1%) as mobile-phase modifier. Separation was conducted under the following gradient for LC–ESI(+)-MS: 0 min 15% (B); 0–1 min 30% (B); 1–1.3 min from 30% to 48% (B); 1.3–5.5 min from 48% to 82% (B); 5.5–5.8 min from 82% to 99% (B); 5.8–6 min 99% (B); 6–6.1 min from 99% to 15% (B); 6.1–7.5 min 15% (B) (+0.5 min preinjection steps). For LC–ESI(−)-MS, the following gradient was used: 0 min 15% (B); 0–1 min 30% (B); 1–1.3 min from 30% to 48% (B); 1.3–3.05 min from 48% to 62% (B); 3.05–3.2 min from 62% to 99% (B); 3.2–3.6 min 99% (B); 3.6–3.7 min from 99% to 15% (B); 3.7–4.6 min 15% (B) (+0.5 min preinjection steps). A sample volume of 1 μL and 3 μL was used for ESI(+) and ESI(−) platforms, respectively. Sample temperature was maintained at 4°C.

The ESI source and MS parameters were: sheath gas pressure, 60 arbitrary units; aux gas flow, 25 arbitrary units; sweep gas flow, 2 arbitrary units; spray voltage ESI(+), 3.6 kV; spray voltage ESI(−), −3.0 kV; capillary temperature, 300°C; aux gas heater temperature, 370°C; MS1 mass range, *m*/*z* 200–1700; MS1 resolving power, 140,000 FWHM (*m*/*z* 200). For selected samples, MS/MS spectra were collected for TG analyzed with ESI(+) platform to obtain molecular species-level information. In this case, the mass spectrometer was operated under the following conditions: MS1 mass range, *m*/*z* 200–1700; MS1 resolving power, 35,000 FWHM (*m*/*z* 200); number of data-dependent scans per cycle, 3; MS/MS resolving power, 17,500 FWHM (*m*/*z* 200), and normalized collision energy of 20%.

Quality control

Quality control was assured by (i) randomization of the actual samples within the sequence, (ii) injection of quality control (**QC**) pool samples at the beginning and the end of the sequence and between each 10 actual samples, (iii) analysis of procedure blanks, (iv) checking the peak shape and the intensity of internal standard added prior to injection.

Data processing

Samples were processed via MS-DIAL 4.24 as before [1,2]. TAG and FA species were annotated using in-built LipidBlast mass spectral library in MS-DIAL. Isotopologues of annotated TAGs were processed via MRMPROBS [3] and isotopic enrichment calculated using in-house Python scripts and IsoCor [4]. FA composition of the TAGs (or the main contributor within the family of isomers) can be deduced from the MS/MS spectra, thus allowing the assessment of TAG acyl composition. Free fatty acids after TAG hydrolysis were processed directly via MRMPROBS and IsoCor.

Table S1. ^{2}H enrichment of various FA moieties in TAG

	STD	HF	HF+F	HF+PIO	HF+PIO+F	HF+MSDC	HF+MSDC+F
FA 12:0	0.52±0.05	0.12±0.02 *	0.07±0.01 *	0.08±0.01 *	0.11±0.02 *	0.15±0.03 *	0.06±0.00 *
FA 14:0	0.86±0.01	0.48±0.02 *	0.49±0.02 *	0.52±0.02 *	0.53±0.02 *	0.53±0.03 *	0.53±0.03 *
FA 14:1	0.77±0.01	0.29±0.07 *	0.18±0.06 *	0.45±0.02 *	0.41±0.05 *	0.47±0.07 *	0.24±0.04 *
FA 16:0	0.65±0.01	0.33±0.02 *	0.35±0.02 *	0.38±0.02 *	0.41±0.02 *	0.39±0.03 *	0.42±0.02 *a
FA 16:1	0.56±0.02	0.33±0.01 *	0.25±0.04 *	0.34±0.01 *	0.31±0.03 *	0.33±0.02 *	0.28±0.03 *
FA 17:0	0.45±0.01	0.21±0.03 *	0.23±0.03 *	0.21±0.03 *	0.27±0.02 *	0.25±0.03 *	0.27±0.04 *
FA 18:0	0.59±0.01	0.28±0.02 *	0.20±0.03 *	0.18±0.03 *	0.26±0.04 *	0.25±0.03 *	0.30±0.03 *
FA 20:1	0.52±0.06	0.14±0.03 *	0.14±0.02 *	0.12±0.01 *	0.12±0.01 *	0.11±0.00 *	0.15±0.04 *

Using LC-MS analysis, a total of 44 individual FA moieties enriched in deuteria were detected in TAG hydrolysates. Only 8 that were present in all the groups were considered. Data were transformed using cube root function. Data are means ± SEM; n = 8; *, significantly different vs. STD ($p \leq 0.05$, t-test); a, significantly different vs. HF ($p \leq 0.05$, one-way ANOVA).

Figure S1. Palmitate oxidation in eWAT

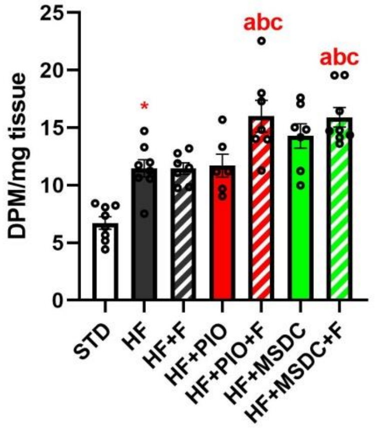

Palmitate oxidation was assessed in fragments of eWAT dissected from mice fed different diets using [1-14C]-palmitate (Perkin Elmer, USA; 0.33 µCi/ml) as described before, in the absence of AICAR [5]. Data are means ± SEM (n = 6 - 8). *, significantly different from STD ($p \leq 0.05$, t-test). a, significantly different vs. HF; b, significantly different vs. HF+F; c, significantly different vs. HF+PIO ($p \leq 0.05$, one-way ANOVA).

Figure S2. Quantification of the complexes of mitochondrial respiratory chain in eWAT

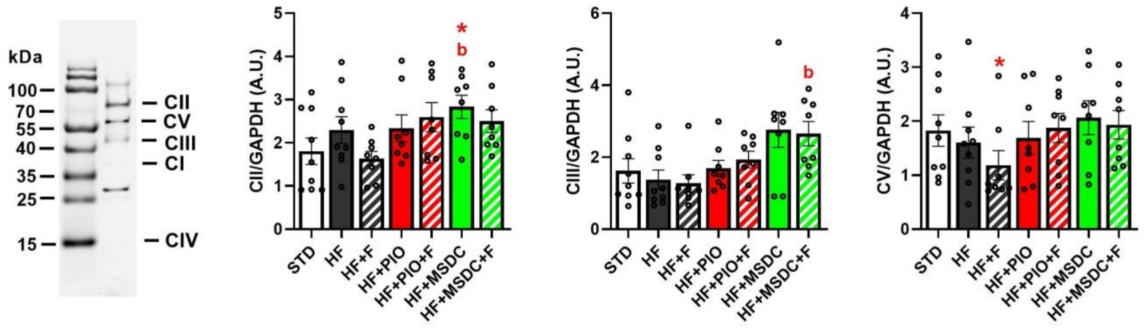

The content of complexes of mitochondrial respiratory chain was evaluated using Western blotting and extracts of eWAT, similarly as before [6]. Briefly, fragments of eWAT were homogenized in lysis buffer under liquid nitrogen, sonicated and centrifuged at 18 000 x g for 10 minutes at 4°C. Protein in the supernatant was determined using bicinchoninic acid assay, and aliquots (10 µg protein) analysed using 10% Tris-glycine-SDS polyacrylamide gels. Blots were stained using Total OXPHOS Blue Native WB Antibody Cocktail (MS603, Abcam, 1:250), which included antibodies against the following protein subunits: Ndufa9 (complex I), Sdha (complex II), Uqcrc2 (complex III), Cox4 (complex IV), Atp5a1 (complex V), as illustrated on the blot on the left with PageRuler Prestained Protein Ladder (10 – 170 kDa; Thermoscientific, Rockford, IL, USA) markers and eWAT homogenate prepared from STD-fed mice. Signals were quantified using the Odyssey IRImaging Systems (Li-Cor Biosciences, Lincoln, NE, USA) and signals on different blots were compared using an internal standard. Due to low intensity of bands corresponding to the complex I and complex IV subunits, only the content of complex II (**CII**), complex III (**CIII**) and complex V (**CV**) was evaluated. An

antibody specific for glyceraldehyde-3-phosphate dehydrogenase (1:1 000, Cell Signalling, MA, USA) was used to control sample loading. Results are expressed in arbitrary units (**A.U.**). Data are means ± SEM, n = 8. *, significantly different from STD ($p \leq 0.05$, t-test). b, significantly different vs. HF+F ($p \leq 0.05$, one-way ANOVA).

References

1. Tsugawa, H.; Cajka, T.; Kind, T.; Ma, Y.; Higgins, B.; Ikeda, K.; Kanazawa, M.; VanderGheynst, J.; Fiehn, O.; Arita, M. MS-DIAL: data-independent MS/MS deconvolution for comprehensive metabolome analysis. *Nat Methods* **2015**, *12*, 523-526, doi:10.1038/nmeth.3393.
2. Paluchova, V.; Oseeva, M.; Brezinova, M.; Cajka, T.; Bardova, K.; Adamcova, K.; Zacek, P.; Brejchova, K.; Balas, L.; Chodounska, H., et al. Lipokine 5-PAHSA Is Regulated by Adipose Triglyceride Lipase and Primes Adipocytes for De Novo Lipogenesis in Mice. *Diabetes* **2020**, *69*, 300-312, doi:10.2337/db19-0494.
3. Tsugawa, H.; Kanazawa, M.; Ogiwara, A.; Arita, M. MRMPROBS suite for metabolomics using large-scale MRM assays. *Bioinformatics* **2014**, *30*, 2379-2380, doi:10.1093/bioinformatics/btu203.
4. Millard, P.; Letisse, F.; Sokol, S.; Portais, J.C. IsoCor: correcting MS data in isotope labeling experiments. *Bioinformatics* **2012**, *28*, 1294-1296, doi:10.1093/bioinformatics/bts127.
5. Kus, V.; Prazak, T.; Brauner, P.; Hensler, M.; Kuda, O.; Flachs, P.; Janovska, P.; Medrikova, D.; Rossmeisl, M.; Jilkova, Z., et al. Induction of muscle thermogenesis by high-fat diet in mice: association with obesity-resistance. *Am J Physiol Endocrinol Metab* **2008**, *295*, E356-367, doi:10.1152/ajpendo.90256.2008.
6. Flachs, P.; Adamcova, K.; Zouhar, P.; Marques, C.; Janovska, P.; Viegas, I.; Jones, J.G.; Bardova, K.; Svobodova, M.; Hansikova, J., et al. Induction of lipogenesis in white fat during cold exposure in mice: link to lean phenotype. *Int J Obes (Lond)* **2017**, *41*, 372-380, doi:10.1038/ijo.2016.228.
